# Zinc Peroxide-Mediated In Situ Forming Hydrogels for Endogenous Tissue Regeneration

**DOI:** 10.34133/bmr.0238

**Published:** 2025-08-12

**Authors:** Yeonjeong Kim, Kyung Min Park

**Affiliations:** ^1^ Department of Bioengineering and Nano-Bioengineering, Incheon National University, Incheon 22012, Republic of Korea.; ^2^ Research Center for Bio Materials & Process Development, Incheon National University, Incheon 22012, Republic of Korea.

## Abstract

Bioactive hydrogels have garnered considerable attention for endogenous tissue regeneration owing to their affordability, minimal regulatory barriers, and ability to harness the body’s intrinsic healing potential. Recently, inorganic-ion-releasing hydrogels have been developed as bioactive matrices, promoting wound healing and tissue repair through external cellular stimulation. Among various therapeutic inorganic ions, zinc ions (Zn^2+^), in particular, play essential roles in wound healing by modulating cell proliferation and angiogenesis and facilitating tissue remodeling. Numerous strategies have been developed to fabricate Zn^2+^-releasing biomaterials; however, these methods often encounter challenges, including complex fabrication processes, rapid ion release, and limited mechanical stability. To address these challenges, we developed a novel Zn^2+^-releasing bioactive hydrogel (Zn-Gel) as a bioactive matrix that supported wound healing via a zinc peroxide (ZnO_2_)-mediated cross-linking reaction. Zn-Gel was fabricated by combining thiolated gelatin with ZnO_2_ solutions, forming a hydrogel with controllable Zn^2+^ release kinetics that depended on ZnO_2_ concentration and enabled sustained release of Zn^2+^ for up to 14 d. Zn-Gel demonstrated excellent cytocompatibility and tissue compatibility in both in vitro and in vivo studies. Interestingly, Zn-Gel accelerated wound healing by promoting cell proliferation, blood vessel formation, hair follicle formation, and collagen deposition. Therefore, Zn-Gel holds great potential as an advanced bioactive material for wound healing and tissue regeneration.

## Introduction

Polymeric hydrogels have garnered increasing attention in biomedical applications owing to their excellent biocompatibility, customizable physical and chemical properties, and structural similarity to the native extracellular matrix (ECM) [1]. Among these, in situ cross-linkable hydrogels, which can undergo sol–gel phase transitions through physical or chemical cross-linking reactions, are particularly promising for tissue regeneration [2]. These hydrogels offer several advantages, including facile encapsulation of therapeutic agents, for example, cells, drugs, and genes; minimally invasive application at target sites; and adaptation to irregular defects [3]. Because of these unique properties, in situ cross-linkable hydrogels have been extensively used as therapeutic vehicles or implants for tissue regeneration [4,5].

An emerging trend in polymeric hydrogel design is developing bioactive matrices that support endogenous tissue regeneration, a process known as in situ tissue regeneration. This approach uses bioactive hydrogels to stimulate surrounding tissues through physical, chemical, and biological signals, enhancing the body’s intrinsic healing capabilities (e.g., endogenous cell and tissue infiltration, immune modulation, neovascularization, and tissue remodeling) [6]. Recently, in situ tissue regeneration has gained considerable attention as an advanced and cost-effective alternative to traditional tissue engineering, offering reduced regulatory barriers and excellent consistency in quality. Extensive research has focused on developing bioactive materials that promote in situ tissue regeneration by enhancing the microenvironment and recruiting endogenous cells to injury sites through various biophysical and biochemical factors (e.g., topography, mechanical properties, and oxygen tension) [7,8]. Although numerous strategies have been proposed, developing hydrogel materials that effectively support endogenous tissue regeneration remains challenging.

Recently, various metal ions, such as zinc, magnesium, calcium, copper, and silver, have been used as bioactive molecules to enhance in situ tissue regeneration [9]. Evidence indicates that these ions regulate cellular functions by activating ion channels, mediating protein binding, and stimulating the gene expression involved in tissue regeneration [10,11]. In particular, Zn^2+^ has garnered particular interest owing to its ability to enhance wound healing processes, including cell proliferation, blood vessel formation, hair follicle development, and collagen synthesis [12]. Various therapeutic strategies have been developed to create Zn^2+^-releasing biomaterials such as metallic zinc alloys, zinc oxide (ZnO) nanoparticles, and Zn^2+^-based metal–organic frameworks (MOFs) [13,14]. However, these approaches often face limitations, such as complex fabrication, rapid ion release, and low mechanical stability [15]. For instance, ZnO nanoparticles are known to exhibit rapid ion release, leading to burst effects and potential cytotoxicity. Zn^2+^-based MOFs, while effective in encapsulating Zn^2+^, typically require complex and multistep synthesis. Such challenges hinder their practical application in tissue regeneration, highlighting the need for alternative platforms that enable controllable, sustained Zn^2+^ release with facile fabrication and physiological stability. Consequently, developing advanced hydrogels that are easy to manufacture and provide controlled Zn^2+^ release remains an urgent need.

To address these limitations, we utilized ZnO_2_ as both a hydrogel cross-linking initiator and a long-term Zn^2+^ release source. Upon decomposition, ZnO_2_ releases Zn^2+^ and hydrogen peroxide (H_2_O_2_). The generated H_2_O_2_ induces disulfide bond formation between thiolated gelatin (GtnSH), enabling gelation under physiological conditions. In addition, the generated Zn^2+^ forms coordination bonds with thiol groups, which govern the prolonged release of Zn^2+^. This strategy not only stabilizes the hydrogel structure but also regulates the sustained release of Zn^2+^, supporting tissue regeneration.

**Fig. 1.**
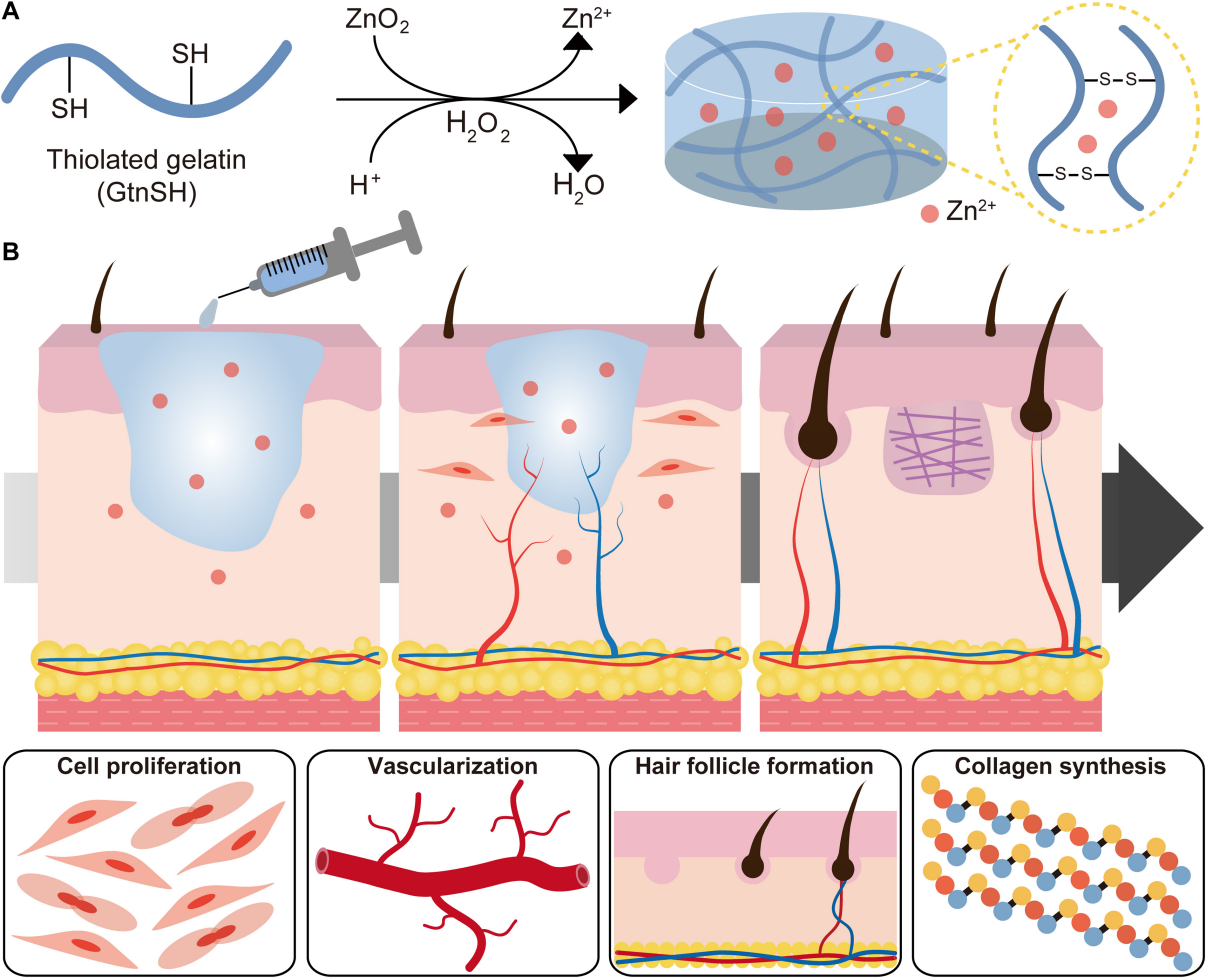
Schematics of in situ cross-linkable Zn-Gel fabrication. (A) Hydrogels are fabricated through ZnO_2_-mediated disulfide bond formation. (B) Therapeutic effects of Zn-Gel on wound healing.

Herein, we present a new type of in situ cross-linkable Zn^2+^-releasing hydrogel (Zn-Gel) with controlled Zn^2+^ release and enhanced bioactivity to facilitate endogenous tissue regeneration. Bioactive hydrogels were easily fabricated by mixing GtnSH and ZnO_2_ solutions (Fig. 1A). Our in situ forming hydrogel system enabled simultaneous hydrogel cross-linking and Zn^2+^ encapsulation. These bioactive hydrogels exhibited outstanding cell and tissue compatibility, demonstrating controllable and sustained Zn^2+^ release. Zn-Gel matrices promote cell proliferation, neovascularization, collagen deposition, and hair follicle formation during skin wound healing and tissue regeneration (Fig. 1B). To our knowledge, this is the first in situ forming hydrogel developed via a ZnO_2_-mediated cross-linking reaction, with potential applications in wound healing and tissue regeneration.

## Materials and Methods

### Materials

To synthesize and characterize GtnSH, we obtained the following materials from reputable sources: gelatin type A (from porcine skin, less than 300 bloom) and anhydrous dimethyl sulfoxide (DMSO) from Sigma-Aldrich (Saint Louis, MO), 2-iminothiolane hydrochloride (Traut’s reagent, TR) and deuterium oxide (D_2_O) from Sigma-Aldrich (Saint Louis, MO), dialysis membranes with a molecular cutoff of 3.5 kDa from Spectrum Laboratories (Rancho Dominguez, CA), 1 N hydrochloric acid (HCl) solution from Daejung (Siheung, Gyeonggi), and 5,5′-dithio-bis(2-nitrobenzoic acid) (Ellman’s reagent) from Thermo Scientific (Rockford, IL). For hydrogel fabrication, we obtained ZnO_2_ from Alfa Aesar (Haverhill, MA). Dulbecco’s phosphate-buffered saline (DPBS) was provided by Gibco (Grand Island, NY), and Tris-HCl (1 M, pH 8.0) was supplied by Invitrogen (Grand Island, NY).

For the in vitro cytocompatibility test, we sourced human dermal fibroblasts (HDFs) from Lonza (Walkersville, MD). The essential cell culture components, including Dulbecco’s modified Eagle medium (DMEM), newborn calf serum (NBCS), penicillin/streptomycin (P/S), and 0.25% trypsin–ethylenediamine tetraacetic acid, were purchased from Gibco (Grand Island, NY). H_2_O_2_ (30 wt%) was provided by Junsei (Tokyo, Japan). We utilized a WST-1 cell proliferation kit from Roche (Basel, Schweiz) and a live/dead kit from Invitrogen (Grand Island, NY) for cytocompatibility experiments.

For the in vivo wound healing test, we used Tegaderm dressings from 3M Health Care (St. Paul, MN). The immunohistochemistry staining kit and antibodies (anti-Ki67, anti-CD31, and anti-cytokeratin 19) were obtained from Abcam (Cambridge, MA). All chemicals and solvents were used as received without further purification.

### Synthesis and characterization of GtnSH

GtnSH was synthesized by incorporating thiol groups into the gelatin backbone, as described previously [16]. In brief, 500 mg of gelatin was dissolved in 50 ml of anhydrous DMSO at 40 °C under a nitrogen atmosphere. Once the gelatin was completely dissolved, a TR (10 mg/ml) solution in anhydrous DMSO was prepared and added to the gelatin solution. The reaction mixture was allowed to react for 24 h. Following the conjugation reaction, the solution was transferred to a dialysis membrane bag with a molecular weight cutoff of 3.5 kDa and dialyzed for 48 h in a 5 mM HCl solution, followed by an additional 24 h in a 1 mM HCl solution. After dialysis, the polymer solution was frozen at −20 °C, and GtnSH polymers were obtained by lyophilization.

To analyze the chemical structure of GtnSH, proton nuclear magnetic resonance spectroscopy was performed using an Agilent 400-MR spectrometer (Agilent Technologies, CA). GtnSH polymer solutions were prepared by dissolving the polymers in D_2_O at a 10 mg/ml concentration.

To determine the thiol content of GtnSH, Ellman’s assay was carried out following the manufacturer’s instructions. In brief, 3 mg of cysteine and the GtnSH polymer were separately dissolved in 3 ml of deionized water (DIW) at a concentration of 1 mg/ml. Subsequently, a 100-μl aliquot of the GtnSH polymer solution was mixed with 100 μl of Ellman’s reagent and allowed to react for 20 min at room temperature. The absorbance values were then measured at a wavelength of 405 nm using an ultraviolet spectrophotometer (Multiskan EX, Thermo Scientific, Rockford, IL). The thiol content was determined by comparing the absorbance values with a cysteine standard curve.

### Fabrication of Zn-Gel and phase transition time measurement

Zn-Gel was prepared by a simple mixing method, combining GtnSH and ZnO_2_ solutions. For the fabrication of 100-μl hydrogels, GtnSH was dissolved in DPBS solution, while ZnO_2_ was dissolved in 1 M Tris-HCl at pH 8.0. The GtnSH and ZnO_2_ solutions were mixed homogeneously at a 9:1 volume ratio. The final concentrations of GtnSH solutions were 10 wt%, while the ZnO_2_ solutions ranged from 0.125 to 0.5 wt%. For further reference, the specific sample codes and the corresponding concentrations of each component can be found in Table.

**Table.** Fabrication of Zn-Gel

Sample code	Polymer (wt%)	ZnO_2_ (wt%)	H_2_O_2_ (mM)
Z0	10	0.000	10
Z0.125	10	0.125	10
Z0.25	10	0.250	10
Z0.5	10	0.500	10

The vial-tilting method was employed to determine the hydrogels’ phase transition time. Using the described procedure, a 100-μl volume of the Zn-Gel formulation was prepared in a 2-ml glass vial. The sol–gel transition time was determined as the point at which the solution no longer flowed, indicating the completion of gelation and the formation of a solid.

### Rheological analysis of Zn-Gel

To investigate the mechanical properties of the hydrogels, rheological analysis was conducted using a rheometric fluid spectrometer (DHR-1, TA Instruments, New Castle, DE). Zn-Gel (200 μl) was carefully placed onto the center of the rheometer plate, with a 20-mm plate geometry applied to establish contact with the hydrogel samples, maintaining a uniform gap of 600 μm. All measurements were conducted at 37 °C, and a solvent trap filled with DIW was employed to minimize solvent evaporation during testing.

Dynamic time sweep tests were performed on hydrogels containing various concentrations of ZnO_2_, with or without 10 mM H_2_O_2_. The measurements were carried out at a constant strain of 0.1% and a frequency of 0.1 Hz. In addition, frequency sweep tests were conducted depending on angular frequencies (0.1 to 100 rad/s) at a constant strain of 0.1%. Furthermore, the shear-rate-dependent viscosity was measured by varying the shear rate from 0.01 to 1,000 s^−1^.

### In vitro hydrogel degradation

To assess the in vitro degradation behavior of Zn-Gel, a degradation study was conducted. The hydrogels (100 μl) were prepared by fabricating them within a 1-ml syringe and subsequently incubated for 1.5 h in a CO_2_ incubator to stabilize them. The hydrogels were then transferred to 1.5-ml microtubes and incubated with either 200 μl of DPBS or a collagenase solution (0.005 mg/ml) at 37 °C. At specific time points (30 min and 1, 3, 6, 9, 24, 48, and 72 h), the media from the microtubes were carefully removed, and the hydrogels were weighed to determine their remaining weight (*W*_d_). Fresh media were then added to the microtubes. The remaining weight of the hydrogels was calculated using the following equation:

$$\text{Weight of hydrogel}\;\left(\%\right)=\left(W_{d}/W_{i}\right)\times 100$$ (1)

where *W*_i_ is the weight of the initial hydrogels and *W*_d_ is the remaining hydrogels’ weight.

### The surface morphology of Zn-Gel

To analyze the surface morphology and elemental composition of Zn-Gel, scanning electron microscopy (SEM) coupled with energy-dispersive spectroscopy (EDS) analysis (SEM–EDS) was performed using a JSM 7001F microscope (JEOL, Tokyo, Japan); 200-μl hydrogel samples with varying ZnO_2_ concentrations (ranging from 0 to 0.5 wt%) were prepared within 1-ml syringes for this analysis. The syringes were then frozen at −20 °C for 18 h. After freezing, the hydrogels were cut and subjected to freeze-drying. The surface of the hydrogels was coated with a layer of gold to enhance conductivity, enabling visualization of the porous structure. SEM–EDS measurements were conducted to observe the surface morphology and perform elemental mapping analysis of the hydrogels.

### Zn^2+^ and H_2_O_2_ measurements

The release of Zn^2+^ from the hydrogels was quantified using the zinc-ligand binding assay. Hydrogel samples (20 μl) with varying ZnO_2_ concentrations (ranging from 0.125 to 0.5 wt%) were prepared and immersed in 500 μl of sterile DIW in a 48-well plate. The plate was then placed in a CO_2_ incubator at 37 °C with 5% CO_2_. At predetermined time points (1, 3, 5, 7, 10, and 14 d), the leachates were carefully collected, and fresh media were added to the wells. The concentrations of released Zn^2+^ from the hydrogels were determined using the zinc assay kit (Sigma-Aldrich, Saint Louis, MO), following the manufacturer’s protocol.

To evaluate the release behavior of H_2_O_2_ from the hydrogels, the Cu(II)–neocuproine spectrophotometric method was employed (Fig. S1). Like in the Zn^2+^ release study, hydrogel samples were prepared as described above; 500 μl of DPBS buffer was incubated with the hydrogels in a 48-well plate at 37 °C in a CO_2_ incubator. All of the DPBS buffer was collected at each predetermined time point, and an equal volume of fresh medium was added to the wells. For the Cu(II)–neocuproine assay, 50 μl of 0.01 M DPBS, hydrogel eluate (sample solution), 0.01 M copper sulfate (CuSO_4_) solution, and 0.01 M neocuproine solution were sequentially added to a 96-well plate. The absorbance of the resulting solution was measured at 450 nm using a microplate reader. The amount of H_2_O_2_ released was calculated by comparing the absorbance values to a H_2_O_2_ standard curve ranging from 0 to 1,000 μM.

### The cytocompatibility of Zn-Gel

To evaluate the cytocompatibility of the hydrogels, HDFs were cultured in DMEM supplemented with 10% NBCS and 1% P/S. The cells were maintained at 37 °C and 5% CO_2_ under standard culture conditions. The GtnSH polymers and ZnO_2_ were sterilized using 15 min of ultraviolet light irradiation to perform cytocompatibility assays. All solutions were prepared using sterilized DPBS and filtered using a syringe filter with a 0.2-μm pore size. HDFs (1.5 × 10^4^ cells/well) were precultured in a 48-well plate (SPL Life Sciences, Korea) with 500 μl of high-glucose DMEM supplemented with 10% NBCS and 1% P/S. Hydrogels (20 μl) with varying ZnO_2_ concentrations (ranging from 0 to 0.5 wt%) were fabricated and placed in a separate 48-well plate containing 500 μl of medium for 24 h. After incubation, the cell culture medium was removed, and 500 μl of diluted hydrogel eluate (a 1:1 volume ratio of cell culture medium and hydrogel eluate) was added and incubated for 24 h at 37 °C in a CO_2_ incubator. The morphology and viability of the cells were assessed using a live/dead assay and optical microscopy (Nikon Eclipse TS100, Nikon, Japan) as well as a WST-1 assay. For fluorescence imaging of cellular morphology, cells were incubated with the hydrogel eluate as described above and treated with a mixture of 2 μM calcein-AM (acetomethoxy derivate of calcein) and 4 μM ethidium homodimer-1 for 15 min at 37 °C. The fluorescence images were acquired using a confocal microscope (LSM 900, Zeiss, Oberkochen, Germany).

### In vivo subcutaneous implantation

All animal experiments were conducted in compliance with the guidelines of the Incheon National University Institutional Animal Care and Use Committee (INU-ANIM-2020-13). To demonstrate the tissue compatibility of Zn-Gel, 6-week-old female C57BL/6N mice were used. The mice were housed under standard conditions for 7 d and randomly divided into 3 groups, with 3 mice in each group: control (hydrogel nontreated group), Z0 (Zn^2+^-free group), and Z0.5 (Zn^2+^-releasing group). The dorsal region of the mice was shaved and sterilized with 70% ethanol and povidone. The hydrogels were fabricated in a 1-ml syringe with a 5-mm diameter and subcutaneously implanted into the dorsal region of the mice. After complete degradation of the hydrogels (on day 21), the animals were sacrificed, and major organs, including the heart, liver, spleen, lungs, and kidneys, were harvested. All samples were fixed in 10% neutral buffered formalin solution for 7 d.

### In vivo critical defect model

An in vivo animal study was conducted to investigate the wound healing effect of Zn-Gel. Female C57BL/6N mice aged 6 to 8 weeks were used for the experiments. The mice were housed under standard conditions for 7 d and randomly divided into 2 groups, with 3 mice in each group: Z0 and Z0.5. After inducing anesthesia using isoflurane, the dorsal area of the mice was shaved, and full-thickness round wounds with a diameter of 8 mm were created on their backs. A volume of 50 μl of Z0 or Z0.5 hydrogels was prepared in an 8-mm mold and applied to the wounds. Subsequently, the wounds were covered with Tegaderm, a transparent film dressing from 3M. Following the surgery, all mice were given intraperitoneal injections of gentamicin and ketoprofen and were housed separately in cages. The healing wounds were photographed at predetermined times (0, 3, 7, 11, and 14 d). The wound area was calculated using the ImageJ software (National Institutes of Health, Bethesda, MD). Additionally, the wound closure ratio (%) was determined using the following equation:

$$\text{Wound closing area}\;\left(\%\right)=\left(A_{0}-A_{i}\right)/A_{0}\times 100$$ (2)

where *A*_0_ is the wound area on day 0 and *A_i_* is the wound area on a predetermined day.

Each group was harvested and histologically analyzed at 7 and 14 d postsurgery. All animal experiments were performed according to the guidelines of the Incheon National University Institutional Animal Care and Use Committee (INU-ANIM-2020-13).

### Histological analysis

Each group was sacrificed at 7 and 14 d postsurgery, and histological analysis was performed. The explants were fixed with a neutral buffered 10% formalin solution. The tissue samples underwent a series of steps, including dehydration, clearing, and paraffin infiltration. Gradually increasing ethanol concentrations (75% to 100%) were used to remove water from the tissue. Subsequently, ethanol was replaced with xylene, and paraffin infiltrated the tissue at 58 °C. Paraffin blocks were then trimmed (10 μm) and sectioned (4 μm) using a microtome. Each paraffin section was placed on an adhesive slide glass and dried for 24 h to remove any remaining water. Hematoxylin and eosin staining and immunohistochemistry staining for Ki67, CD31, and cytokeratin 19 were performed to investigate the wound healing effect. Masson’s trichrome staining was employed to detect collagen deposition. The animal study was conducted following a protocol approved by the Incheon National University Institutional Animal Care and Use Committee (INU-ANIM-2020-13).

### Statistical analysis

Data are expressed as mean ± standard deviation, with at least 3 samples. Statistical analyses of the data were performed by a *t* test using GraphPad Prism 5 (GraphPad Software Inc., La Jolla, CA). The statistical significance for all tests was set at *P < 0.05, **P < 0.01, and ***P < 0.001.

## Results and Discussion

### Zn-Gel fabrication and controllable physicochemical properties

**Fig. 2.**
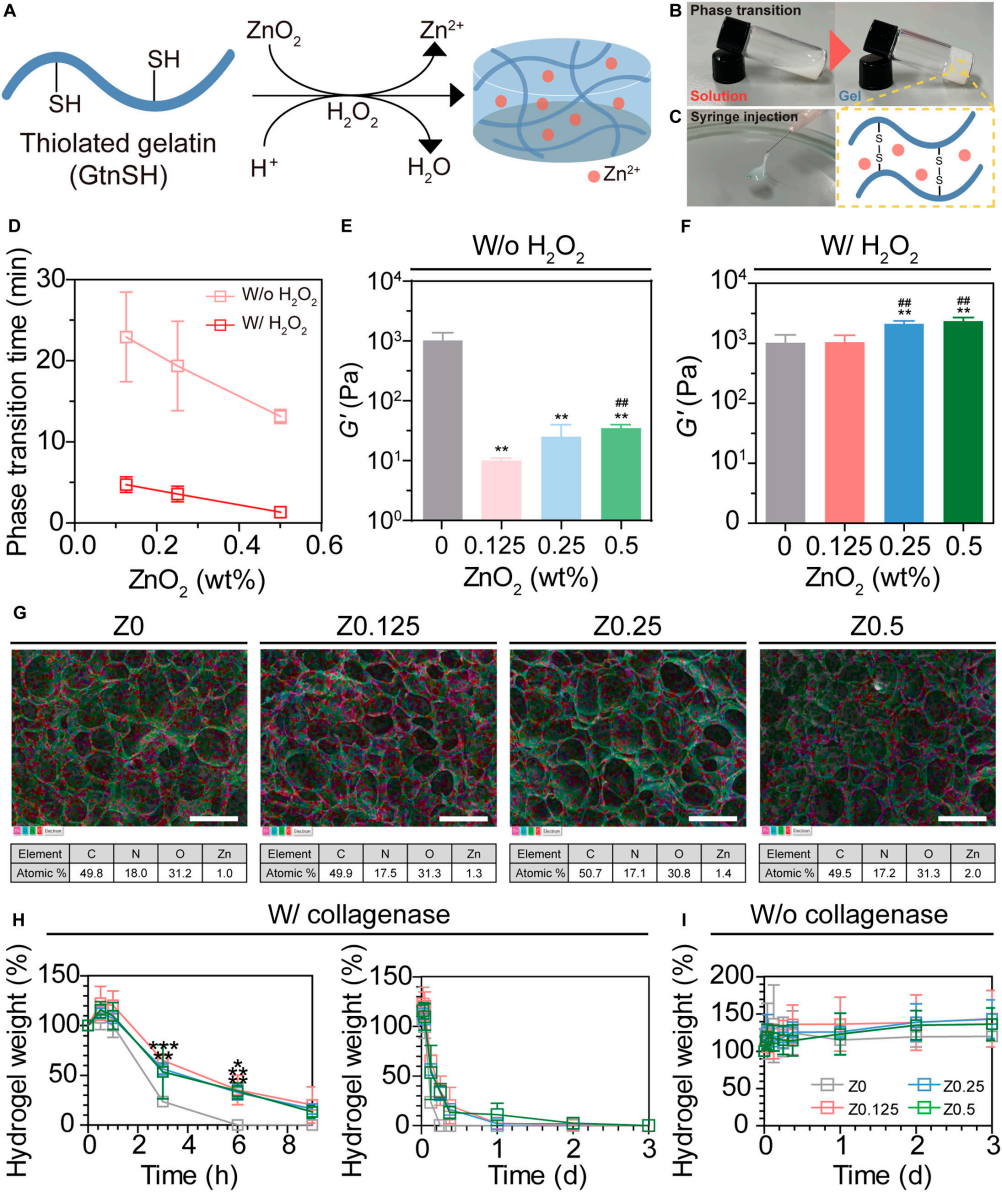
Synthesis and characterization of the Zn-Gel. (A) Schematic representation of gel formation. (B) Digital images of the sol–gel transition and (C) hydrogel injection. (D) Phase transition time depending on ZnO_2_ concentration and addition of H_2_O_2_. Rheological analysis depending on ZnO_2_ concentration (E) without H_2_O_2_ solution and (F) with H_2_O_2_ solution. (G) Porous structure and atomic % of the hydrogels using scanning electron microscopy coupled with energy-dispersive spectroscopy (SEM–EDS) analysis. In vitro hydrogel degradation ratio using (H) collagenase type II and (I) Dulbecco’s phosphate-buffered saline (DPBS). The results in (E), (F), (H), and (I) are shown as average value ± SD (*n* = 3). * indicates significant difference from Z0 (*P < 0.05; **P < 0.01; ***P < 0.001). ## indicates significant difference from Z0.125 (^##^*P* < 0.01). Scale bars represent 250 μm.

We hypothesized that introducing thiol groups into the gelatin backbone enables the fabrication of Zn-Gel by utilizing H_2_O_2_ in ZnO_2_-mediated cross-linking reactions. ZnO_2_ reacts with acidic media to generate H_2_O_2_ as an intermediate, which ultimately decomposes into water. During hydrogel formation, H_2_O_2_ facilitates network formation by inducing disulfide bonds between the GtnSH polymers through oxidative cross-linking (Fig. 2A). Although ZnO_2_ is widely used as an antibacterial reagent and in drug delivery, its potential for fabricating Zn-Gel has not been explored.

First, GtnSH was synthesized for hydrogel formation. Gelatin, a natural polymer derived from collagen, was selected as the polymer backbone for its cellular activity, biocompatibility, and biodegradability [17]. GtnSH was synthesized by conjugating TR to the gelatin backbone (Fig. S2) as described previously [18]. Zn-Gel was prepared by mixing GtnSH, ZnO_2_, and H_2_O_2_ solutions. Disulfide bond formation resulted in homogeneously structured Zn-Gel hydrogels, incorporating Zn^2+^ within the matrix and demonstrating a sol–gel phase transition (Fig. 2B). The injectability of Zn-Gel was also tested using a 26-gauge (26G) syringe needle, demonstrating smooth hydrogel flow through the needle (Fig. 2C). These results demonstrate that Zn-Gel, which can be easily fabricated and used as an injectable hydrogel, holds considerable potential for biomedical applications in wound healing, tissue engineering, and drug delivery systems and applications requiring minimally invasive delivery methods.

Phase transition time is crucial for the effective application and injection of materials at target sites. A slow phase transition may cause material diffusion from the target site, whereas a rapid transition increases the risk of syringe clogging and complicates the filling of irregular wound defects [19]. To optimize phase transition time, we measured it across different ZnO_2_ concentrations (0.125 to 0.5 wt%) using the vial-tilting method. The phase transition time decreased as the ZnO_2_ concentration increased, ranging from 23 min at lower concentrations to 13 min at higher concentrations. Additionally, adding a H_2_O_2_ solution (final concentration of 10 mM) during hydrogel formation reduced the phase transition time from 5 min to 1 min and 30 s (Fig. 2D). These results indicate that higher ZnO_2_ concentrations accelerate disulfide bond formation owing to increased intermediate H_2_O_2_ production.

Zn-Gel exhibited a controllable phase transition time by adjusting the ZnO_2_ concentration within an optimal range of 2 to 10 min. For instance, a short transition time of approximately 3 to 5 min is beneficial for rapid wound closure, offering rapid protection against external contaminants. Conversely, a longer transition time of approximately 7 to 10 min enables more controlled and gradual delivery, which is advantageous for applications requiring the sustained release of therapeutic agents. This adjustability in phase transition time enhances Zn-Gel’s versatility for diverse wound regeneration applications.

The mechanical properties of biomaterials are crucial for hydrogel stability, enabling them to withstand external stimuli and maintain structural integrity [10]. To assess these properties, we conducted a rheological analysis using dynamic time sweeps. Higher ZnO_2_ concentrations (0.125 to 0.5 wt%) and the addition of H_2_O_2_ (final concentration of 10 mM) resulted in increased *G*′ values compared with those of hydrogels without H_2_O_2_. Specifically, the *G*′ values of hydrogels with H_2_O_2_ were 2,150 Pa (Z0.125), 2,440 Pa (Z0.25), and 2,640 Pa (Z0.5), whereas those for hydrogels without H_2_O_2_ were significantly lower at 10 Pa (Z0.125), 30 Pa (Z0.25), and 40 Pa (Z0.5) (Fig. 2E and F). To further evaluate hydrogel stability and injectability, frequency sweep and viscosity measurements were performed. Zn-Gel maintained a stable *G*′ across the frequency range and exhibited a decrease in viscosity with increasing shear rate, indicating structural stability under dynamic mechanical conditions and a favorable flow behavior (Fig. S3). These results confirm that Zn-Gel possesses tunable mechanical strength, stable viscoelastic properties, and injectability, supporting its applicability in biomedical settings requiring both durability and ease of administration.

Microporous biomaterials support cell infiltration, migration, vessel invasion, and inflammatory cell recruitment [20]. SEM–EDS analysis was conducted to evaluate the porosity of the hydrogels. The SEM images of Zn-Gel revealed interconnected porous structures (Fig. 2G), suggesting their potential for tissue regeneration applications owing to their ability to facilitate cell infiltration and migration. Additionally, the zinc concentration increased (0.125 to 0.5 wt%) with increasing atomic zinc percentage (Z0, 0.99%; Z0.125, 1.33%; Z0.25, 1.38%; and Z0.5, 1.98%), indicating the higher Zn^2+^ content within the hydrogel at increased ZnO_2_ concentrations.

The degradability of hydrogels is essential for supporting new tissue formation, as controlled degradation ensures structural integrity and sustains therapeutic effects [21]. We assessed the degradability of Zn-Gel by measuring its weight after incubation in DPBS and type II collagenase for 3 d. Hydrogels incubated in DPBS remained swollen over 3 d (day 3 weights: Z0, 119.8 mg; Z0.125, 143.6 mg; Z0.25, 143.0 mg; and Z0.5, 136.2 mg), whereas those incubated with collagenase exhibited complete degradation within the same period (day 1 weights: Z0, 0 mg; Z0.125, 2.5 mg; Z0.25, 2.0 mg; and Z0.5, 11.2 mg; day 3 weights: all samples degraded to 0 mg). These results indicate that Zn-Gel retains its proteolytic degradability after thiol functionalization and Zn^2+^ addition. Additionally, the degradation rate can be adjusted by varying the ZnO_2_ concentration (Fig. 2H and I). These findings demonstrate that Zn^2+^ delays hydrogel degradation by modulating metalloproteinase activity through interactions with their catalytic sites [22]. These findings also highlight the potential of Zn^2+^ to enhance hydrogel stability and functionality for biomedical applications.

### Sustained release of Zn^2+^

Zn^2+^, the second most abundant trace element in the human body, is essential in wound healing and tissue regeneration [23]. During wound healing, zinc contributes to various functions, including cell migration and proliferation, immune modulation, blood vessel formation, and hair follicle stem cell recruitment [24]. Consequently, various Zn^2+^-releasing biomaterials have been developed for these applications, such as Zn-based MOFs, ceramic materials, nanoparticles, and hydrogels. These materials are used in drug delivery systems, wound healing applications, and tissue regeneration because they promote antimicrobial activity, modulate immune responses, and support angiogenesis. However, these approaches have limitations, such as complex fabrication processes and rapid Zn^2+^ release [25,26]. Thus, we developed a novel hydrogel that is easy to fabricate and enables the controlled release of Zn^2+^ during hydrogel formation.

**Fig. 3.**
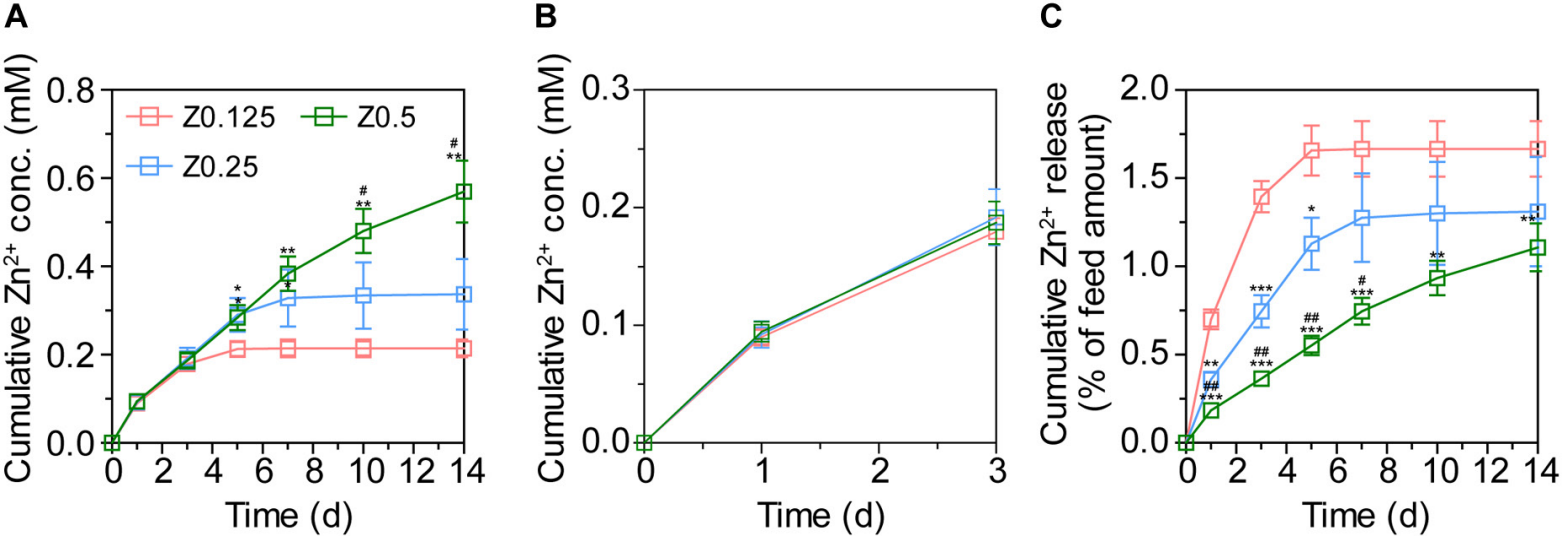
Zn^2+^ release behaviors depending on ZnO_2_ concentrations. Zn^2+^ cumulative release graphs for (A) 14 d and (B) 3 d. (C) % of feed amount for 14 d. The results in (A) to (C) are shown as average value ± SD (*n* = 3). * indicates significant difference from Z0.125 (*P < 0.05; **P < 0.01; ***P < 0.001). # indicates significant difference from Z0.25 (^#^*P* < 0.05; ^##^*P* < 0.01).

To quantify Zn^2+^ concentrations released from the hydrogel matrices, we performed a Zn^2+^ detection assay. Zn-Gel was fabricated with varying ZnO_2_ concentrations (0.125, 0.25, and 0.5 wt%) and incubated in DIW for 14 d. Figure 3A and B show the cumulative release profiles of Zn^2+^ from the hydrogel matrices. The released Zn^2+^ concentrations increased with higher ZnO_2_ concentrations (Z0.125, 0.09 mM; Z0.25, 0.092 mM; and Z0.5, 0.094 mM after 24 h; and Z0.125, 0.21 mM; Z0.25, 0.34 mM; and Z0.5, 0.57 mM after 14 d), indicating that the ZnO_2_ concentration increased with increasing concentration of released Zn^2+^. Given that daily Zn^2+^ release exceeding 0.1 mM can be cytotoxic, our results confirmed that the Zn^2+^ released from our hydrogels remained below this threshold, minimizing potential cytotoxicity.

The release behavior of Zn^2+^ is critical, as short-term release may not provide sufficient Zn^2+^ to the injury site, whereas excessive release can lead to cytotoxicity. Consequently, we quantified the cumulative Zn^2+^ release as a percentage of the initial amount loaded (Fig. 3C). Our results showed that only 1.1% to 1.7% of the total Zn^2+^ in the hydrogel was released over the study period (Z0.125, 0.7%; Z0.25, 0.35%; and Z0.5, 0.18% after 1 d; and Z0.125, 1.7%; Z0.25, 1.3%; and Z0.5, 1.1% after 14 d). This controlled release was attributed to the formation of Zn^2+^–thiol complexes, which enabled Zn^2+^ to be gradually released over time [27]. This mechanism provides a sustainable supply of Zn^2+^, essential for effective wound healing while preventing cytotoxicity.

Overall, the developed Zn-Gel provides a sustained supply of Zn^2+^ over the long term. Throughout the 14 d, the hydrogels consistently released Zn^2+^ at noncytotoxic levels. These findings suggest that our hydrogel addresses the limitations of existing Zn^2+^-releasing biomaterials, offering simple fabrication and prolonged release, rendering it suitable for various biomedical applications.

### Cytocompatible hydrogels

The investigation of cytocompatibility is essential to ensure the safety and suitability of biomaterials for clinical applications [28]. We conducted in vitro cell studies using HDFs to assess the cytocompatibility of the hydrogels. HDFs, the predominant cell type in the dermal layer [29], are crucial in wound remodeling, migration, proliferation, and the secretion of growth factors, cytokines, and ECM components [30,31]. Therefore, we used HDFs for cell culture.

**Fig. 4.**
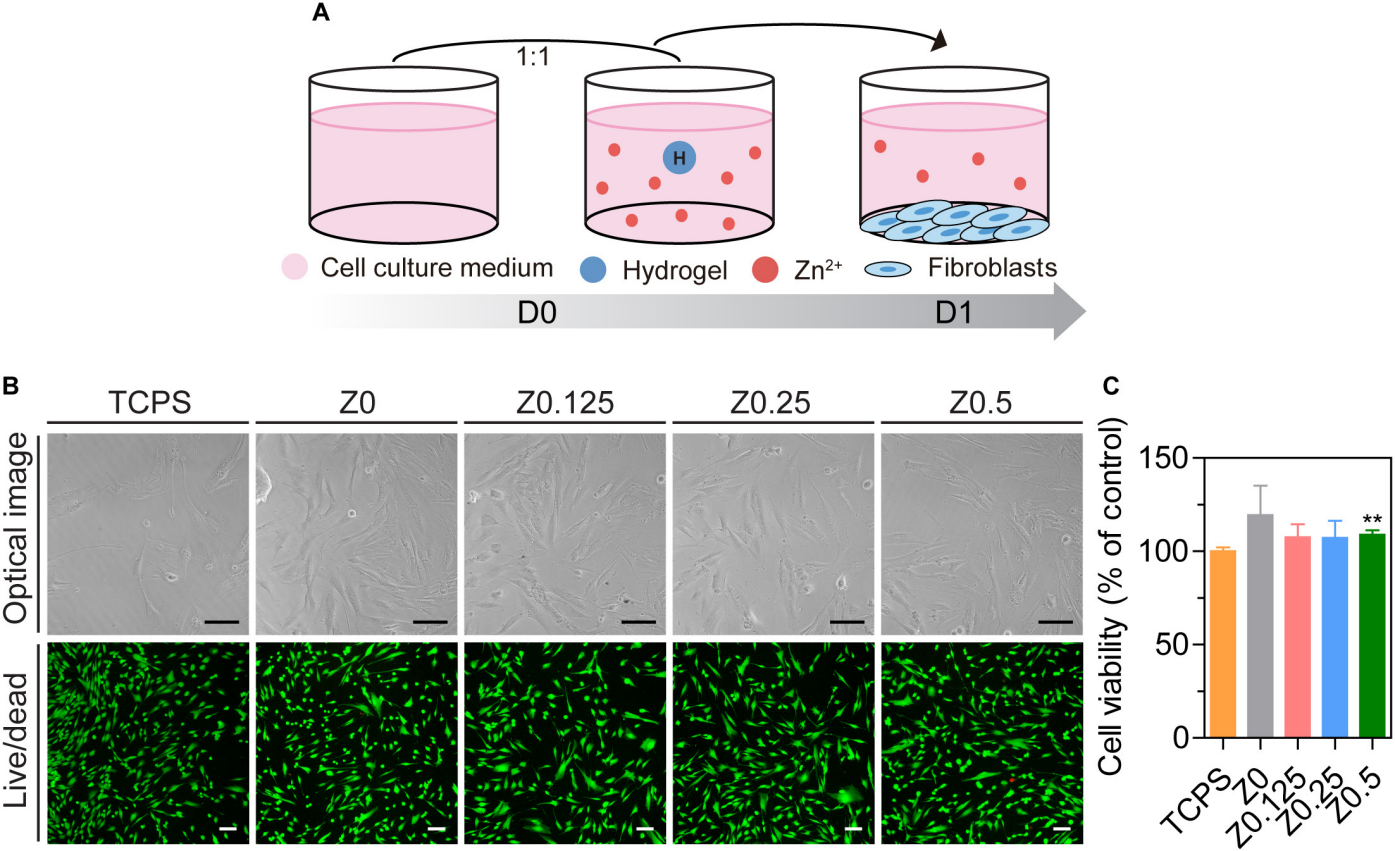
In vitro cytocompatibility of Zn-Gel. (A) Schematic representations of the cytotoxicity test by hydrogel eluate culture. (B) Optical images and fluorescence microscopic images of human dermal fibroblasts (HDFs) cultured with hydrogel eluate for 1 d (live cells in green; dead cells in red). (C) Cell viability assessment of HDFs (% of control; tissue culture polystyrene [TCPS] at day 1) using the WST-1 assay. The results in (C) are shown as average value ± SD (*n* = 4). ** indicates significant difference from the control group (TCPS) (**P < 0.01). Scale bars represent 100 μm.

HDFs were cultured with diluted hydrogel eluates for 24 h to assess cell viability and morphology (Fig. 4A). A control group without hydrogel eluate was cultured on tissue culture polystyrene (TCPS) for comparison. Optical microscopy and live/dead assay images showed that most cells remained viable and displayed normal morphology when exposed to hydrogel eluates (Fig. 4B). Cell viability was quantified using the WST-1 assay (Fig. 4C), with all hydrogel groups demonstrating outstanding cell viability (Z0, 124%; Z0.125, 105%; Z0.25, 112%; and Z0.5, 109% relative to TCPS). Notably, the Z0.5 group exhibited a significant increase in cell viability compared with TCPS. This enhancement, particularly in the Z0.5 group, was likely due to the presence of Zn^2+^, which was vital in cellular processes such as proliferation, differentiation, and survival. Zn^2+^ can enhance cellular activity by interacting with cellular proteins and enzymes, serving as a cofactor in numerous biochemical reactions [32]. Additionally, Zn^2+^ influences gene expression during cell growth and repair [33], contributing to the observed increase in cell viability.

Our study demonstrated the outstanding cytocompatibility of Zn-Gel, highlighting its potential for clinical applications. Through in vitro cytocompatibility assessment, we optimized the hydrogel samples and selected representative groups. The Zn^2+^-free group (Z0) released H_2_O_2_ without Zn^2+^, whereas the Zn^2+^-containing group (Z0.5) released both Zn^2+^ and H_2_O_2_ at equivalent concentrations (10 mM). The selection of the Z0.5 group was based on its superior cell viability and sustained Zn^2+^ release over an extended period, providing a favorable environment for tissue regeneration.

### Biodegradable and tissue-compatible acellular matrices

Biodegradability is a crucial factor in evaluating the efficacy of materials for biomedical applications. When implanted or injected into the human body, nonbiodegradable hydrogels or scaffolds may pose risks of permanent retention, potentially leading to long-term physiological and psychological complications [34]. Degradation is essential for facilitating cell stretching and proliferation and supporting tissue growth in tissue engineering. Therefore, hydrogel scaffolds are designed to degrade and be cleared from the body with minimal impact on the surrounding tissues and organs, as cells form functional tissues [10]. This process is crucial for the success of tissue engineering and regenerative medicine. Additionally, the tissue compatibility of hydrogels and their degradation products is critical for tissue regeneration, requiring thorough evaluation to ensure clinical suitability [35].

**Fig. 5.**
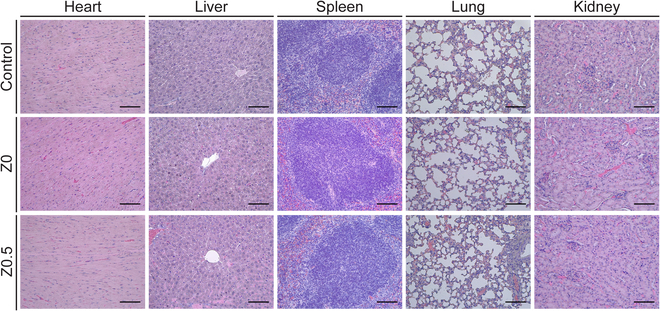
In vivo tissue compatibility test. Histological sections of major organs (heart, liver, spleen, lung, and kidney) stained with hematoxylin and eosin (H&E) (*n* = 3). Scale bars represent 100 μm.

In vivo subcutaneous implantation was performed to assess the biodegradability and tissue compatibility of the hydrogels [34]. Two types of hydrogels (Z0 and Z0.5) were fabricated and subcutaneously implanted into mice. Zn-Gel gradually degraded over 3 weeks. After complete hydrogel degradation, hematoxylin and eosin staining was conducted on the major organs, including the heart, liver, spleen, lungs, and kidneys. Figure 5 demonstrates that the Zn-Gel-treated organs showed normal tissue structures without pathological symptoms. Furthermore, no inflammatory cell infiltration was observed in the major organs. These findings indicate that Zn-Gel exhibits excellent in vivo biodegradability and tissue compatibility, highlighting its potential as a wound healing material.

### Enhanced wound healing with improved cell proliferation, vascular recruitment, hair follicle development, and collagen synthesis

Inorganic-ion-releasing bioactive materials are known to enhance tissue regeneration by regulating and stimulating cellular functions [36]. Among these ions, Zn^2+^ is crucial in wound healing, cell proliferation, vascular cell recruitment, hair follicle development, and collagen regeneration [37,38]. Based on these properties, we hypothesized that our Zn-Gel could promote tissue regeneration by sustainably releasing Zn^2+^ at the injury site. Zn^2+^ promotes wound healing via multiple biological mechanisms. First, it acts as a cofactor for enzymes involved in DNA synthesis and cell proliferation [39]. Second, Zn^2+^ promotes endothelial cell migration and stimulates the expression of angiogenic factors such as vascular endothelial growth factor A [40]. Third, it influences the viability of stem cells in hair follicles [37]. Finally, Zn^2+^ enhances the cross-linkage of collagen and the regulation of ECM formation [32].

**Fig. 6.**
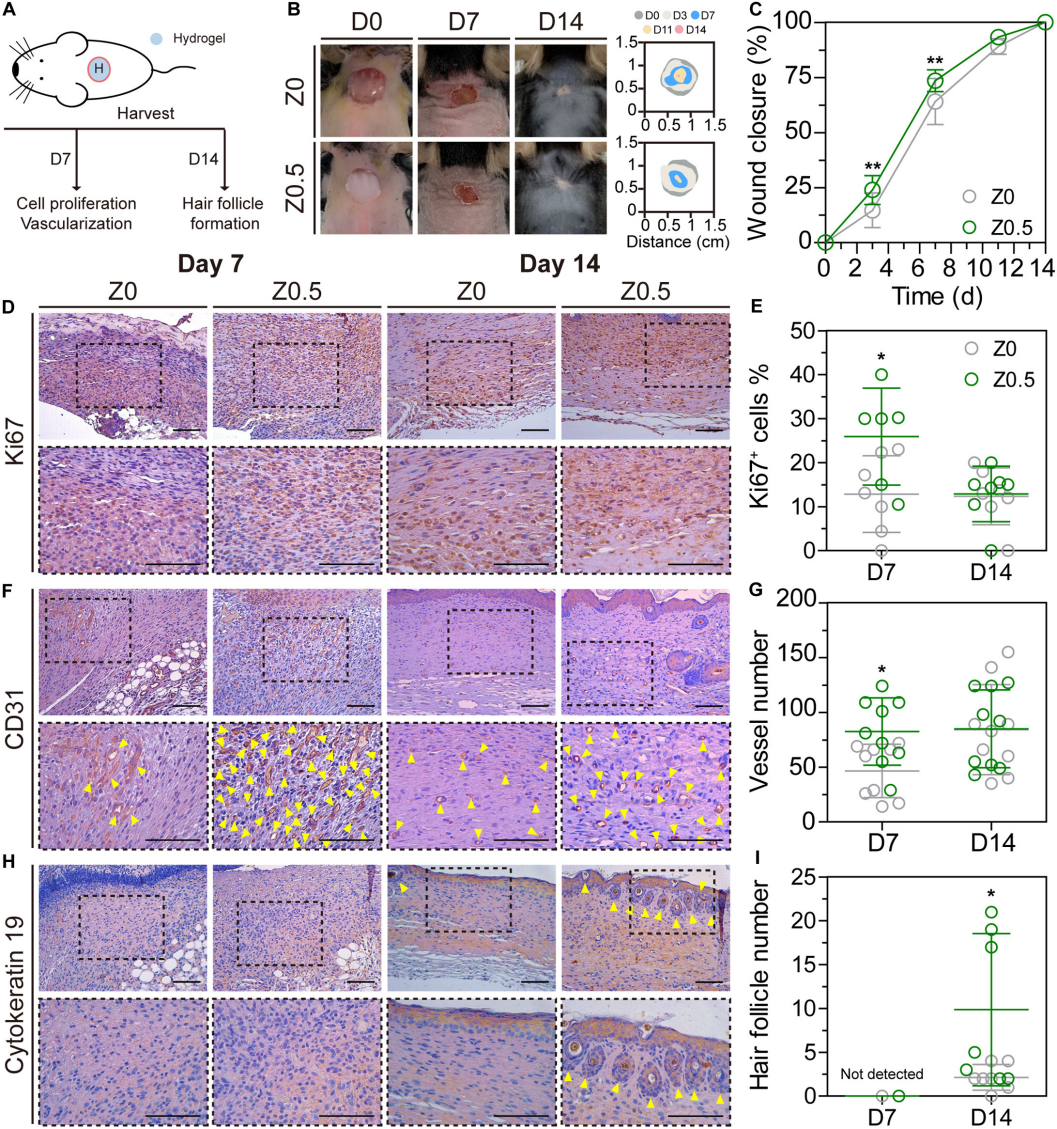
In vivo wound healing effects of Zn-Gel. (A) Schematic representations of the critical defect model. (B) Digital images of mouse skin defect models at days 0, 7, and 14. (C) Quantitative analysis of wound closure. Images of immunohistochemistry staining and quantification for Ki67 (D and E), CD31 (F and G), and cytokeratin 19 (H and I) at 7 and 14 d. Arrowheads (▲) indicate blood vessel structures and hair follicles. The results in (C) are shown as average value ± SD (*n* = 3 to 15). The results in (E), (G), and (I) are shown as average value ± SD (*n* = 6 to 9). * indicates significant difference from Z0 (*P < 0.05; **P < 0.01). Scale bars represent 100 μm.

We applied the hydrogels to mouse critical skin defect models to evaluate the wound healing effect of Zn-Gel (Fig. 6A). To evaluate the effect of Zn^2+^ within the hydrogel system, experimental groups were selected based on differences in zinc content. Two types of hydrogels (Z0 and Z0.5) were applied to 8-mm defects in the dorsal area of the mice for 2 weeks. The digital images in Fig. 6B show the hydrogel treatments of the wound defect models. The wound area was measured at specific time points to evaluate wound healing. The images revealed an increased wound closure rate in the Z0.5 group compared with that in the Z0 group (Z0, 14%, and Z0.5, 23% at day 3; Z0, 64%, and Z0.5, 73% at day 7; and Z0, 100%, and Z0.5, 100% at day 14). Quantitative analysis of wound closure confirmed that the Z0.5 group exhibited a faster reduction in wound area than the Z0 group (Fig. 6C), suggesting that Zn-Gel accelerates skin wound healing.

Wound healing is a complex process involving multiple factors. Effective regeneration depends on cell proliferation, vascular formation, and follicle regeneration [41,42]. To further examine the histological characteristics of the regenerated tissues, mouse skin samples were collected on days 7 and 14 and stained for specific markers, including Ki67 (a marker of cell proliferation [43]), CD31 (a marker of vascular endothelial cells [44]), and cytokeratin 19 (a marker of hair follicles involved in enhancing skin functionality [45]).

Ki67 staining revealed significantly enhanced cell proliferation in the Z0.5 group compared with that in the Z0 group (Z0, 12%, and Z0.5, 25% at day 7; and Z0, 14%, and Z0.5, 15% at day 14) (Fig. 6D and E). Additionally, the Z0.5 group exhibited greater blood vessel formation than the Z0 group (Z0, 47 vessels/mm^2^, and Z0.5, 83 vessels/mm^2^ at day 7; and Z0, 80 vessels/mm^2^, and Z0.5, 84 vessels/mm^2^ at day 14) (Fig. 6F and G). Notably, hair follicle structures were observed in the Z0.5-treated group (not detected on day 7; Z0, 2 follicles/area, and Z0.5, 10 follicles/area on day 14) (Fig. 6H and I and Fig. S4). During wound healing, cell proliferation and vascular formation showed early enhancement, as reflected by the increased staining on day 7, followed by a decrease on day 14. Conversely, hair follicle regeneration improved in the later stages of wound healing, with no follicle structures observed on day 7. Hair follicle development was confirmed by day 14. These results demonstrate the in vivo therapeutic effect of Zn-Gel, indicating that it accelerates wound healing by promoting cellular proliferation, vascular formation, and hair follicle development. Consequently, our hydrogel holds promise for wound healing and tissue regeneration applications.

**Fig. 7.**
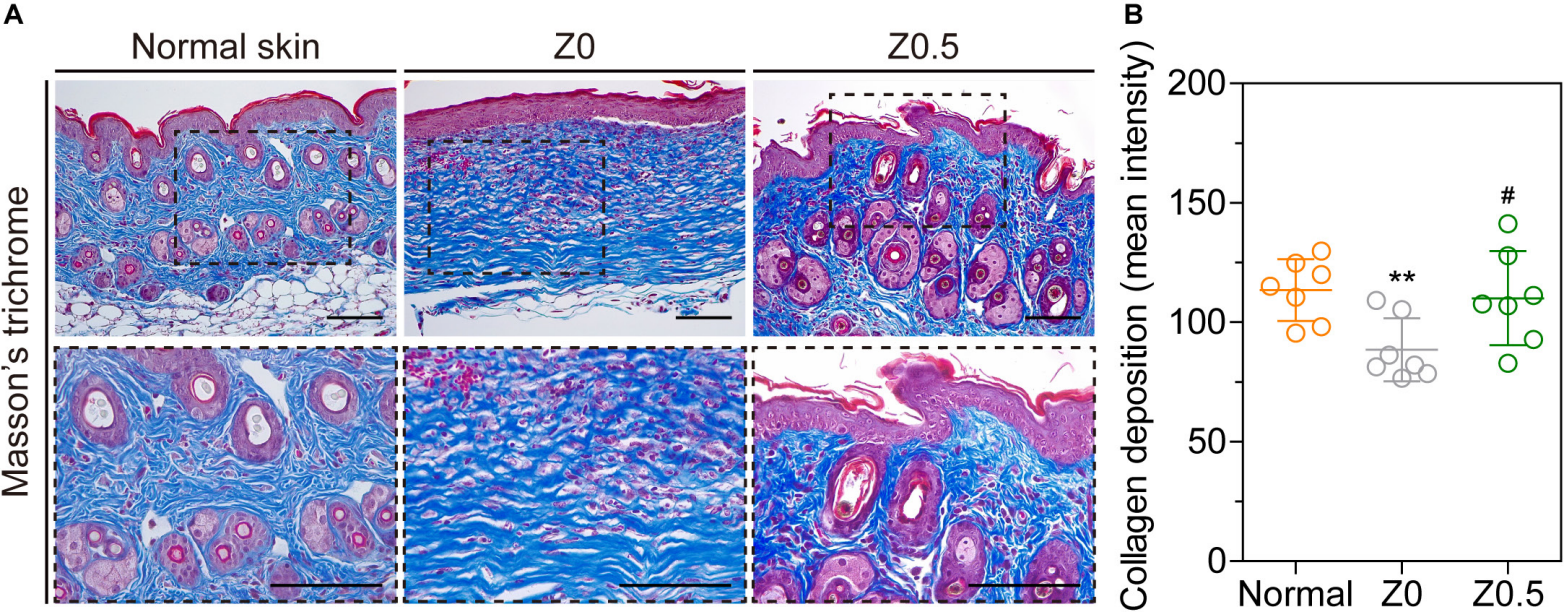
In vivo collagen regeneration effect of Zn-Gel. (A) Histological sections stained with Masson’s trichrome. (B) Quantification of collagen deposition. The results in (B) are shown as average value ± SD (*n* = 3). * indicates significant difference from the normal (**P < 0.01), and # indicates significant difference from Z0 (^#^*P* < 0.05). Scale bars represent 100 μm.

During the remodeling phase, proper collagen deposition is crucial for enhancing the tissue tensile strength [46]. To assess collagen maturation, we used Masson’s trichrome staining, a histological technique that differentiates tissue components (mainly collagen). In this method, muscle fibers and the cytoplasm showed various shades of red, and collagen fibers displayed blue or green hues [47]. The results revealed a blue regenerated skin tissue, confirming collagen regeneration in all tissue samples (Fig. 7A).

Quantitative analysis of collagen deposition showed that the Z0.5 group exhibited collagen synthesis levels comparable to those of the normal skin tissue and higher than those of the Z0 hydrogel group (normal skin, 113 mean intensity/field; Z0, 88 mean intensity/field, and Z0.5, 110 mean intensity/field at day 14) (Fig. 7B). This finding suggests that Zn-Gel may enhance wound remodeling and achieve structural similarity to normal skin by facilitating ECM remodeling. It is known that Zn^2+^ can also inhibit the activity of endogenous enzymes and protect collagen fibers from degradation [48]. Additionally, Zn^2+^ can improve the expressions of collagen types 1 and 3, promoting collagen deposition [49]. This interaction highlights the essential role of Zn^2+^ in collagen production, contributing to the structural integrity and functionality of connective tissues [50]. Although the specific mechanisms by which Zn^2+^ influences wound healing are not fully understood, studies suggest that Zn^2+^ promotes wound healing and regulates its progression. Overall, these results confirm that Zn-Gel can facilitate wound healing by enhancing collagen synthesis through sustained Zn^2+^ release in vivo. Therefore, Zn-Gel may be valuable for wound healing and tissue regenerative medicine.

## Conclusion

This study developed a bioactive Zn-Gel with controllable mechanical properties, sustained Zn^2+^ release, and long-term in vivo stability. The controlled release of Zn^2+^ supports wound healing by promoting cell proliferation, angiogenesis, hair follicle development, and collagen synthesis, providing bioactive stimulation to surrounding tissues. Thus, our Zn-Gel shows promise for various biomedical applications, including wound management and tissue regeneration. Based on these findings, Zn-Gel holds promise not only for wound management and tissue regeneration but also for broader biomedical applications. Given the known antibacterial and hemostatic effects of Zn^2+^, the hydrogels were further applied to infected wound models or as a bioactive dressing. Moreover, due to the effects of Zn^2+^ in bone regeneration, the hydrogels may be adapted for orthopedic applications. Future work will focus on enhancing therapeutic efficacy by co-delivering various ions such as Ca^2+^ or Mg^2+^ and applying Zn-Gel in large animal models, such as porcine wounds.

## Ethical Approval

All animal protocols were approved by the Incheon National University Institutional Animal Care and Use Committee (INU-ANIM-2020-13).

## Data Availability

The datasets used and analyzed during the current study are available from the corresponding author on reasonable request.
