# Supplementary material for: Zinc Peroxide-Mediated In Situ Forming Hydrogels for Endogenous Tissue Regeneration
Source: Biomater Res. 2025 Aug 12;29:0238. doi: 10.34133/bmr.0238 (PMC12342684; doi:10.34133/bmr.0238)
Supplement: Supplementary 1 — Figs. S1 to S4 [file bmr.0238.f1.docx]

Supplementary Materials

**Zinc Peroxide-mediated In Situ Forming Hydrogels for Endogenous Tissue Regeneration**

**Yeonjeong Kim^1^, Kyung Min Park^1,2,*^**

^1^Department of Bioengineering and Nano-Bioengineering, Incheon National University, Republic of Korea

^2^Research Center for Bio Materials & Process Development, Incheon National University, Republic of Korea

*Corresponding author

K.M. Park, E-mail: kmpark@inu.ac.kr

**
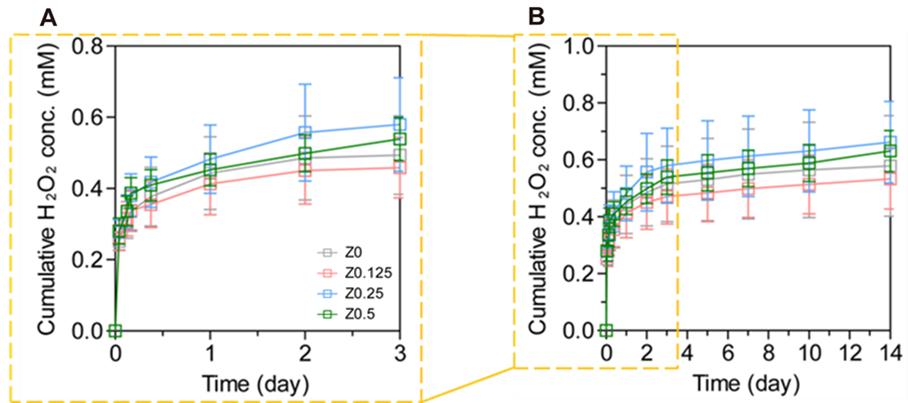
**

Fig. S1. H_2_O_2_ release behavior. (A) H_2_O_2_ cumulative release graph for 3 days and (B) 14 days. The results in (A,B) are shown as the average value ± s.d. (n=6).


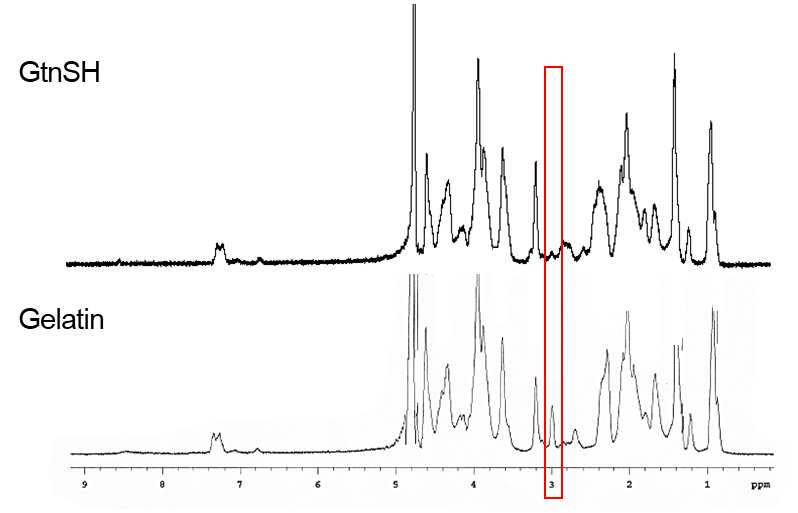


**Fig. S2.** Characterizations of thiolated gelatin (GtnSH). ^1^H-NMR spectra of gelatin and GtnSH.


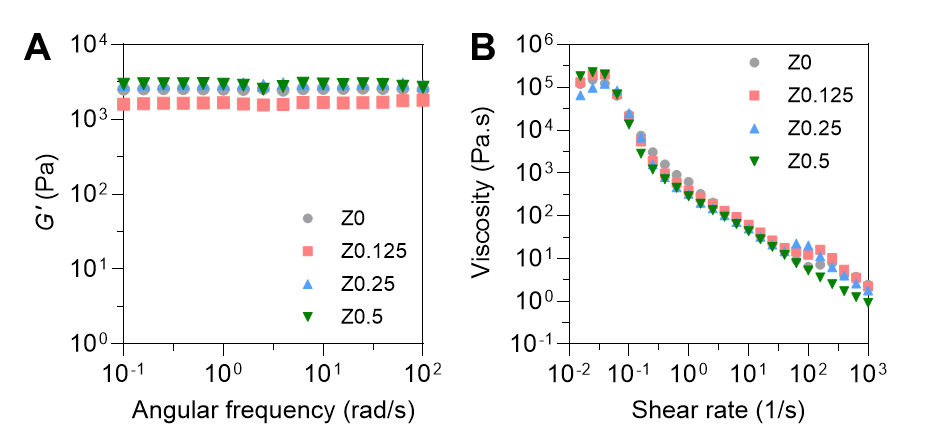


**Fig. S3.** Rheological characterization of Zn-Gel. (A) Frequency sweep curves showing G' depending on angular frequency (0.1–100 rad/s). (B) Viscosity profiles measured over a shear rate range of 0.01-1000 s-1.


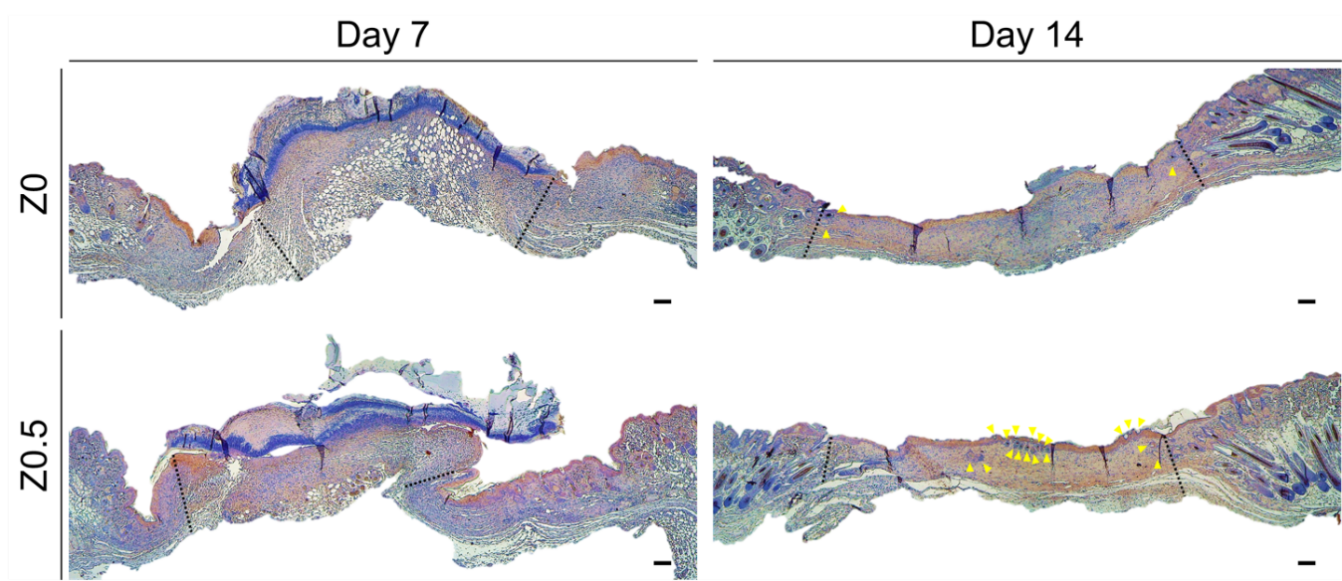


Fig. S4. IHC staining of cytokeratin 19. Whole-mount images of wound sections stained for cytokeratin 19 on days 7 and 14. Yellow arrowheads indicate hair follicles within the regenerated wound area. Scale bars represent 100 μm.
